# Short- and Long-Term Outcomes of Neoadjuvant Chemoradiotherapy Followed by Pancreatoduodenectomy in Elderly Patients with Resectable and Borderline Resectable Pancreatic Cancer: A Retrospective Study

**DOI:** 10.3390/jcm13051216

**Published:** 2024-02-21

**Authors:** Hironobu Suto, Takuro Fuke, Hiroyuki Matsukawa, Yasuhisa Ando, Minoru Oshima, Mina Nagao, Shigeo Takahashi, Toru Shibata, Hiroki Yamana, Hideki Kamada, Hideki Kobara, Hiroyuki Okuyama, Kensuke Kumamoto, Keiichi Okano

**Affiliations:** 1 Department of Gastroenterological Surgery, Faculty of Medicine, Kagawa University, 1750-1 Ikenobe, Miki-cho, Kita-gun, Miki 761-0793, Kagawa, Japan; fuke.takuro@kagawa-u.ac.jp (T.F.); matsukawa.hiroyuki@kagawa-u.ac.jp (H.M.); ando.yasuhisa.rf@kagawa-u.ac.jp (Y.A.); oshima.minoru@kagawa-u.ac.jp (M.O.); nagao.mina@kagawa-u.ac.jp (M.N.); kumamoto.kensuke@kagawa-u.ac.jp (K.K.); okano.keiichi@kagawa-u.ac.jp (K.O.); 2 Department of Molecular Oncologic Pathology, Faculty of Medicine, Kagawa University, 1750-1 Ikenobe, Miki-cho, Kita-gun, Miki 761-0793, Kagawa, Japan; 3 Department of Radiation Oncology, Faculty of Medicine, Kagawa University, 1750-1 Ikenobe, Miki-cho, Kita-gun, Miki 761-0793, Kagawa, Japan; takahashi.shigeo@kagawa-u.ac.jp (S.T.); shibata.toru@kagawa-u.ac.jp (T.S.); 4 Department of Gastroenterology and Neurology, Faculty of Medicine, Kagawa University, 1750-1 Ikenobe, Miki-cho, Kita-gun, Miki 761-0793, Kagawa, Japan; yamana.hiroki@kagawa-u.ac.jp (H.Y.); kamada.hideki@kagawa-u.ac.jp (H.K.); kobara.hideki@kagawa-u.ac.jp (H.K.); 5 Department of Clinical Oncology, Kagawa University, 1750-1 Ikenobe, Miki-cho, Kita-gun, Miki 761-0793, Kagawa, Japan; okuyama.hiroyuki@kagawa-u.ac.jp

**Keywords:** neoadjuvant chemoradiotherapy, pancreatoduodenectomy, pancreatic ductal adenocarcinoma, prognostic nutritional index

## Abstract

**Background**: The efficacy of neoadjuvant chemoradiotherapy (NACRT) followed by pancreatoduodenectomy (PD) in elderly patients with pancreatic ductal adenocarcinoma (PDAC) remains unclear. **Methods**: This retrospective analysis of prospectively collected data examined the effect of NACRT followed by PD in elderly patients with PDAC. A total of 112 patients with resectable (R-) and borderline resectable (BR-) PDAC, who were planned for PD and received NACRT between 2009 and 2022, were assessed. Changes induced by NACRT, surgical outcomes, nutritional status, renal and endocrine functions, and prognosis were compared between elderly (≥75 years, *n* = 43) and non-elderly (<75 years, *n* = 69) patients over two years following PD. **Results**: Completion and adverse event rates during NACRT, nutritional status, renal function, endocrine function over two years postoperatively, and prognosis did not significantly differ between the two groups. Low prognostic index after NACRT and the absence of postoperative adjuvant chemotherapy may be adverse prognostic indicators for elderly patients undergoing NACRT for R- and BR-PDAC. **Conclusions**: Despite a higher incidence of postoperative complications, NACRT followed by PD can be safely performed in elderly patients, resulting in a prognosis similar to that in non-elderly patients.

## 1. Introduction

According to the latest Global Health Statistics for 2023 published by the World Health Organization, global life expectancy has increased to approximately 73.0 years in 2019 and is projected to reach 77.0 years by 2048 [1]. As the average life expectancy continues to rise, the number of pancreatoduodenectomies (PD) performed on elderly patients increases proportionately. With advancements in surgical techniques and perioperative care, PD is increasingly recognized as safe and effective for pancreatic ductal adenocarcinoma (PDAC), even among the elderly [2,3,4,5,6]. However, PD, a highly invasive pancreatic resection technique, has prompted numerous discussions concerning its suitability and significance for elderly patients with pancreatic cancer [4,5,6,7,8,9].

In contrast, preoperative treatment followed by potentially curative pancreatic resection has become the standard of care for cases of resectable (R-) and borderline resectable (BR-) PDAC, per the National Comprehensive Cancer Network (NCCN) guidelines [10]. However, substantial evidence regarding the safety and effectiveness of PD following preoperative therapy in elderly patients with R- or BR-PDAC is lacking. Consequently, debates on upfront surgery in elderly patients may not directly apply to those who have undergone preoperative treatment.

Studies on short-term treatment outcomes in elderly patients with PDAC are available; however, relatively few reports exist on the long-term outcomes and the evolution of patients’ overall health status after PD.

Previously, we have demonstrated the efficacy of neoadjuvant chemoradiotherapy (NACRT) followed by pancreatic resection in elderly patients with R- and BR-PDAC [11]. Notably, our study included various types of pancreatic resections, including distal and total pancreatectomies. PD is more invasive than other pancreatic resections, particularly among elderly patients, because of the risk of postoperative complications and other related factors. Nonetheless, the short- and long-term outcomes of preoperative treatment followed by PD for R- and BR-PDAC among elderly patients remain unknown.

Therefore, this study aimed to evaluate the short- and long-term outcomes in elderly patients who underwent NACRT followed by PD for R- and BR-PDAC and compare them with those of non-elderly patients.

## 2. Materials and Methods

### 2.1. Study Participants

This study included 112 consecutive patients diagnosed with histologically confirmed PDAC who underwent NACRT planned for PD at our hospital between October 2009 and December 2022. All biopsies were performed endoscopically through the stomach or duodenum. No severe complications were observed in any of the patients who underwent biopsy. We conducted NACRT as part of prospective trials only for cases where histological confirmation of PDAC had been obtained. During this time frame, prospective phase II NACRT trials were conducted, utilizing three distinct regimens. Data were methodically collected, and informed consent was obtained from all patients in accordance with our hospital’s institutional protocol prior to commencing the study. The patients were categorized into two groups: those who were ≥75 years (the “elderly group”, *n* = 43) and <75 years (the “non-elderly group”, *n* = 69).

### 2.2. Neoadjuvant Chemoradiotherapy

NACRT was administered in three consecutive prospective phase II trials. The first (University Hospital Medical Information Network Clinical Trials Registry [UMIN-CTR] 000026438) was conducted between September 2009 and May 2016 [12]. Hypofractionated external-beam radiotherapy (30 Gy in 10 fractions) was delivered alongside concurrent S-1 (60 mg/m^2^) given five days a week for two weeks before PD—the “2-week regimen”. The second trial (UMIN-CTR 000035232) was conducted between June 2016 and December 2019, in which external-beam radiotherapy (50 Gy in 25 fractions) was performed, combined with concurrent S-1 (60 mg/m^2^) for five weeks preceding PD—the “5-week regimen” [13]. Finally, the third prospective phase II trial (UMIN-CTR number 000038585) was conducted between January 2020 and December 2022. It involved short-term neoadjuvant hypofractionated chemoradiotherapy (30 Gy in 10 fractions) with Gemcitabine (1000 mg/m^2^) and S-1 (60 mg/m^2^) for two weeks prior to PD—the “2-week S-1 with Gemcitabine regimen”.

S-1 is an orally administered fluoropyrimidine comprising tegafur, a precursor of fluorouracil, and two bioactive modulators. It hinders the activity of dihydropyrimidine dehydrogenase through gimeracil, sustains elevated levels of fluorouracil, and inhibits fluorouracil phosphorylation in the gastrointestinal tract via oteracil potassium. This mechanism effectively mitigates gastrointestinal side effects.

In these NACRT trials, patients with R- and BR-PDAC were enrolled in accordance with the NCCN guidelines [10]. Patients eligible for these trials had Eastern Cooperative Oncology Group performance status (PS) scores of 0–1, were aged 20–85 years, and met specific criteria demonstrating optimal bone marrow reserves and organ function.

Regardless of the presence of cholangitis, preoperative biliary drainage was performed in all patients with obstructive jaundice to prevent liver dysfunction caused by the jaundice before NACRT.

### 2.3. Surgery

All PDs followed the Whipple procedure, and reconstruction was achieved through pancreatojejunostomy using an open surgical approach. The pylorus was resected in all cases of PD. Each patient underwent systematic lymph node dissection.

### 2.4. Follow-Up

Adjuvant chemotherapy after surgery was given unless the patient’s medical condition posed a contraindication. Following the findings of the CONKO-001 trial [14] between 2009 and 2012, Gemcitabine was typically administered to most patients. Since 2013, patients have been prescribed S-1 in accordance with specified treatment protocols based on recommendations from the JASPAC01 trial [15].

Regarding postoperative monitoring, the patients underwent regular check-ups every 2–3 months during the initial year and subsequently every 6 months until disease progression was observed. Enhanced multidetector row computed tomography (MDCT) scan was conducted every 6 months. Whenever necessary, the examination schedule was adjusted to include supplementary tests such as magnetic resonance imaging or fluorodeoxyglucose positron emission tomography (FDG-PET).

### 2.5. Measurement of Results

The variables encompassed the subsequent parameters: sex; age; body mass index (BMI); hemoglobin and hemoglobin A1c (HbA1c) levels; Eastern Cooperative Oncology Group PS; comorbidities such as chronic kidney disease, cardiac disease, diabetes mellitus, and pulmonary disease; use of anticoagulants and steroids; resectability in accordance with the NCCN guidelines [10]; NACRT protocol utilization; completion rate of NACRT protocol; adverse events due to NACRT; white blood cell count, including lymphocytes, neutrophils, monocytes, and platelets; serum albumin level; total cholesterol; total bilirubin level; prognostic nutritional index (PNI); estimated glomerular filtration rate (eGFR); C-reactive protein level (CRP); neutrophil-to-lymphocyte ratio (NLR); lymphocyte-to-monocyte ratio (LMR); platelet-to-lymphocyte ratio (PLR); C-reactive protein-to-albumin ratio (CRP/Alb); carcinoembryonic antigen (CEA) and carbohydrate antigen 19-9 (CA19-9) levels; standardized uptake value (SUV) on FDG-PET; operation duration; blood loss and transfusions; portal vein resection; mortality; complications graded according to the Dindo classification [16]; presence of pancreatic fistula; delayed gastric emptying; length of hospital stay; resection status; positive lymph node; pathological tumor response due to NACRT; TNM stage [17]; adjuvant chemotherapy after surgery; and cause of death.

Overall survival (OS) referred to “the length of time from the start of initial NACRT treatment until death from any cause, with censoring at the date of the last confirmed survival for survivors or at the date of the last confirmed survival before loss to follow-up”. Recurrence-free survival (RFS) referred to “the duration from the time of surgery to the date of relapse or death from any cause”. A thorough assessment of MDCT, tumor markers, and FDG-PET features was performed.

### 2.6. Statistical Analyses

The Fisher’s exact and Mann–Whitney U test or χ2 tests for continuous and categorical variables, respectively, were used to assess the distinctions between the elderly and non-elderly groups. Survival analysis was conducted using the Kaplan–Meier method, and group comparisons were performed using the log-rank test. For continuous variables, cutoff values were determined using receiver operating characteristic curve analysis. The optimal cutoff value was determined by maximizing the Youden index, which considers sensitivity and specificity to achieve the highest classification accuracy [18]. In the multivariate analysis, variables that were identified as potential factors influencing OS (*p* < 0.10) were included in the univariate analysis. This analysis was conducted using a Cox proportional hazards model. Statistical significance was set at a threshold of *p* < 0.05. All statistical analyses were performed using IBM SPSS Statistics for Windows, version 25 (IBM Corp., Armonk, NY, USA).

## 3. Results

Table 1 presents a comparative analysis of baseline characteristics and NACRT outcomes between the elderly and non-elderly groups. Within the entire cohort, 43 (38%) and 69 (62%) patients were aged ≥75 years and <75 years, respectively. The 2-week S-1, 5-week S-1, and 2-week S-1 with gemcitabine NACRT regimens were administered to 42 (38%), 39 (35%), and 31 (28%) patients, respectively. Notably, a significant association existed between the elderly and non-elderly groups with PS scores of 0 or 1 at diagnosis (*p* = 0.002). Concerning the three distinct NACRT regimens, no significant differences were observed between the elderly and non-elderly groups in terms of induction rate, completion rate, or incidence of grade 3 or higher adverse events.

Table 2 summarizes the changes and comparisons of complete blood count, renal function, nutritional markers, inflammatory biomarkers, tumor markers, and SUVmax on FDG-PET before and after NACRT for the elderly and non-elderly groups. Significant differences were observed in neutrophil and platelet counts, albumin levels, and PNI between the elderly and non-elderly groups before NACRT; however, the differences were attenuated after NACRT. Notably, these changes were generally consistent between both groups. Furthermore, the eGFR remained lower in the elderly group before and after NACRT.

Table 3 compares the perioperative and pathological outcomes between the elderly and non-elderly groups in resected cases. According to the Clavien–Dindo system [16], the occurrence of severe complications (Grade ≥ 3) was notably more frequent in the elderly group in comparison to the non-elderly group (29% vs. 13%, *p* = 0.047). Additionally, significant differences were observed between both groups regarding delayed gastric emptying (34% vs. 16%, *p* = 0.037), length of postoperative hospital stay (28 vs. 22 days, *p* = 0.022), and the presence of R0 resection (89% vs. 100%, *p* = 0.009).

### 3.1. Long-Term Evolution of Nutritional Markers, Renal Function, and Endocrine Function in Patients Undergoing Neoadjuvant Chemoradiotherapy Followed by Pancreatoduodenectomy

**Figure 1:**
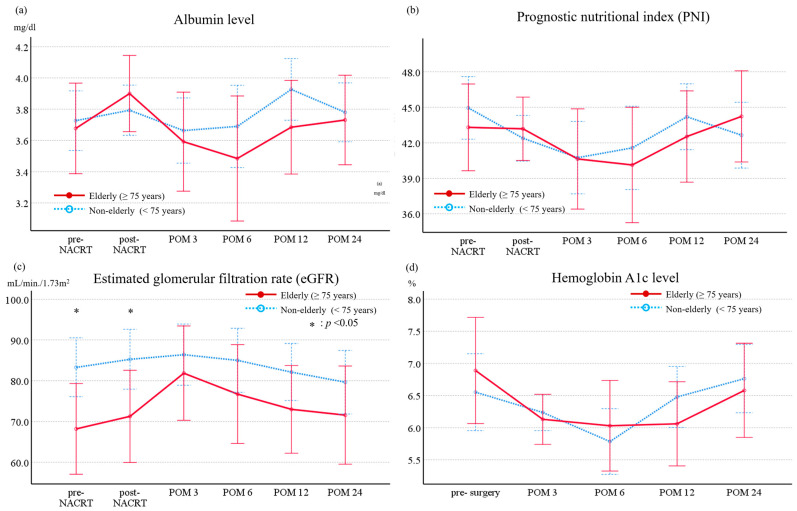
Comparisons of changes in (**a**) albumin level, (**b**) prognostic nutritional index, (**c**) estimated glomerular filtration rate, (**d**) hemoglobin A1c level from before neoadjuvant chemoradiotherapy to 2 years after surgery between the elderly and non-elderly groups. The plots depict the average values, with error bars representing the standard errors of the mean (±2SE).

Figure 1 illustrates the long-term changes in nutritional markers, including albumin levels and PNI; eGFR reflecting renal function; and HbA1c levels indicating endocrine function in patients who underwent NACRT followed by PD. No significant differences were observed in nutritional markers or HbA1c levels between the elderly and non-elderly groups over the observation period from before NACRT to 2 years after surgery. Nevertheless, eGFR levels exhibited a significant decline in the elderly group when compared to the non-elderly group both before and after NACRT (*p* = 0.027 and 0.042, respectively). Moreover, postoperative values were similar between both groups at both time points.

### 3.2. Long-Term Survival

**Figure 2:**
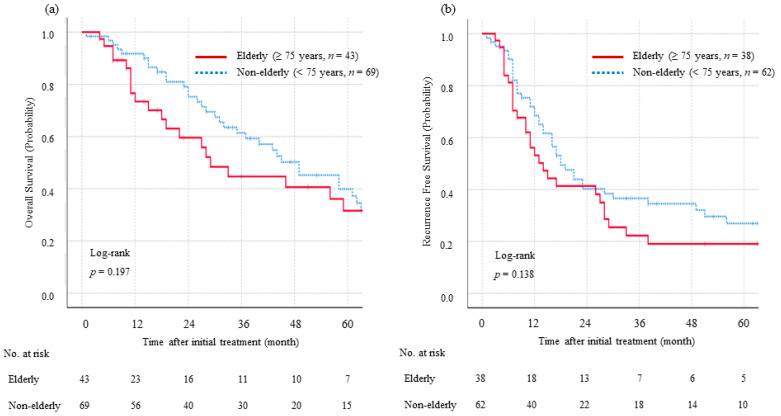
Comparison of overall survival (OS) and recurrence-free survival (RFS) between the elderly and non-elderly groups revealed no statistically significant disparities in either OS or RFS between these two groups. (**a**) No significant differences in OS were observed between the groups in intention-to-treat analyses (*p* = 0.197). (**b**) The elderly group demonstrated an RFS outcome comparable to that of the non-elderly group in resected cases (*p* = 0.138).

The median follow-up duration was 25 months (range: 1–113 months). The elderly cohort had a median OS of 27 months, while the non-elderly cohort had a median OS of 43 months. No significant differences in OS were observed between the groups in intention-to-treat analyses (*p* = 0.197) (Figure 2a). Additionally, the elderly group demonstrated an RFS outcome comparable to that of the non-elderly group in resected cases (*p* = 0.138) (Figure 2b).

Table 4 reveals the univariate and multivariate analyses of prognostic factors for OS in the elderly group using a Cox proportional hazards model. In the univariate analysis, patients with lymphocyte counts post-NACRT ≤ 958/mm^3^ (*p* = 0.024), PNI post-NACRT ≤ 36.9 (*p* = 0.019), lack of postoperative adjuvant chemotherapy (*p* < 0.001), and intraoperative transfusion (*p* = 0.018) exhibited significantly worse OS. However, PNI post-NACRT of ≤ 36.9 (hazard ratio [HR] = 3.95, 95% confidence interval [CI]: 1.06–14.74, *p* = 0.041) and lack of postoperative adjuvant chemotherapy (HR = 9.57, 95% CI: 2.98–30.68, *p* < 0.001) were independent, significant predictors of OS in the multivariate analyses.

## 4. Discussion

Our study is the first to investigate the short- and long-term outcomes of NACRT followed by PD in elderly patients with R- and BR-PDAC using data collected in a prospective manner. We observed no significant differences in terms of NACRT, adverse events, or completion and resection rates between the elderly and non-elderly patient groups, irrespective of the three treatment approaches employed.

The importance of preoperative treatment has been reported in BR-PDAC [22,23,24] and R-PDAC [25], leading to its widespread adoption in various resectability types. Given the well-established relevance of postoperative adjuvant chemotherapy [14,15], three key pillars will define the future of pancreatic cancer management: preoperative treatment, surgery, and postoperative treatment. However, concerns have been raised regarding postoperative treatment, particularly for elderly patients, because of low completion rates and the challenges of providing adequate care due to the invasiveness of surgery and delayed postoperative recovery [4,11]. Thus, preoperative treatment may be crucial to the future of pancreatic cancer treatment for elderly patients. This study revealed a disparity between the postoperative adjuvant chemotherapy completion rates of elderly and non-elderly patients. However, in terms of the rate of adverse events and completion rate of NACRT, both groups exhibited comparable outcomes. In contrast, the lack of adjuvant chemotherapy after surgery was an independent adverse prognostic factor in elderly patients (Table 4). The challenges associated with postoperative recovery in elderly patients can hinder postoperative adjuvant chemotherapy, thereby affecting their prognosis. However, the adverse events and completion rates of NACRT were comparable in both groups. These findings suggest that NACRT can be effectively administered even in elderly patients who may encounter difficulties in completing postoperative adjuvant therapy.

Numerous studies have indicated that elderly and non-elderly patients exhibit similar short-term results and morbidity rates following PD due to recent advancements in perioperative care and surgical techniques [3,6]. Conversely, some reports have demonstrated the heightened occurrence of postoperative morbidities in elderly patients [4,26]. Furthermore, we observed a significantly higher frequency of severe morbidity and delayed gastric emptying, which resulted in prolonged postoperative hospital stays. This emphasizes that issues that appear less significant in non-elderly patients may assume greater importance in elderly individuals. Ballarin et al. [27] indicated that comorbid conditions may be risk factors for morbidity in elderly patients. Here, elderly patients demonstrated significantly lower eGFR levels and a less favorable PS compared with non-elderly patients. Nevertheless, this study provides evidence that NACRT following PD can be safely performed in elderly patients, even in the presence of a heightened morbidity rate and extended hospital stays. Therefore, meticulous patient selection and management are crucial, considering each patient’s unique medical history and background.

Utsumi et al. [7] evaluated long-term changes in nutritional status over one year after PD, which revealed that elderly patients had significantly lower albumin levels than their non-elderly counterparts, suggesting that elderly patients might face a prolonged recovery period and be at risk of malnutrition. Conversely, this study did not reveal differences in nutritional indicators, including albumin and PNI values, between the elderly and non-elderly groups over a two-year postoperative follow-up period. Other research encompassed the analysis of various medical conditions beyond pancreatic cancer, and no disparities were identified between elderly and non-elderly patients in terms of nutritional and immune markers, except for albumin levels. However, our multivariate analysis identified low preoperative PNI as an independent adverse prognostic factor in the elderly group. The association between PNI values and postoperative outcomes extends to various malignancies beyond pancreatic cancer [28,29,30], making it a pivotal topic for further comprehensive research on the dynamics of preoperative and postoperative nutritional status.

In the elderly group, the rate of intraoperative transfusion was significantly higher (Table 3). Transfusions were performed at the discretion of the anesthesiologist, without clear criteria for initiation. However, factors such as the degree of preoperative anemia and the impact on postoperative cardiac function were considered, which may have contributed to the higher incidence of intraoperative transfusion in the elderly group. Likewise, the R0 resection rate in the elderly group was 89%, which was significantly lower than the 100% rate in the non-elderly group. Primarily, for the pancreatic transection margin, additional resection was performed if the intraoperative frozen section diagnosis was positive, aiming for R0 resection. However, in the case of elderly patients, there were no clear criteria, which might have led to a tendency to avoid excessive invasion.

A higher incidence of DGE was observed in the elderly group. In the PDs performed in this study, nasojejunal feeding tubes were placed for early postoperative nutritional support in all cases. Therefore, even during periods when oral intake was not possible due to DGE, enteral nutrition via the feeding tube was administered, and in all cases, conservative management with gastric decompression and fasting led to improvement after DGE. The benefits of early postoperative nutrition have been widely reported [31,32,33], and although this study observed an extended length of hospital stay in the elderly group, these interventions may have contributed to a reduction in postoperative complications [33].

This study had several limitations. First, we retrospectively analyzed prospectively collected data from a single institution. Consequently, the study was limited by the sample size and influenced by historical factors, including potential selection bias and heterogeneity. This prevented us from reaching definitive conclusions. Validation of the findings in larger studies will be required. Additionally, three distinct NACRT regimens were employed in this study; however, NACRT methods should be standardized and analyzed jointly. Moreover, the elderly group was defined as those aged ≥75 years. Thus, the inadequate representation of markedly elderly patients, specifically those aged ≥80 years, hindered a more comprehensive analysis. Finally, our findings mainly apply to elderly patients with R- and BR-PDAC who met the eligibility criteria and can be compared with their younger counterparts in terms of organ function. Nonetheless, this may not be applicable to elderly patients with impaired organ function, who are often ineligible for participation in clinical trials.

## 5. Conclusions

The administration of NACRT followed by PD in elderly patients with R- and BR-PDAC is safe despite the higher incidence of severe complications, delayed gastric emptying, R1 resection, and longer postoperative hospital stays in elderly patients than in non-elderly patients. Moreover, the induction and completion of NACRT are feasible even for elderly patients with postoperative adjuvant therapy complications. Therefore, aggressive treatment approaches, including preoperative therapy, may be considered for elderly patients with well-preserved organ function who meet the eligibility criteria for clinical trials. However, a low PNI after NACRT and the absence of postoperative adjuvant chemotherapy may be adverse prognostic indicators for elderly patients undergoing NACRT for R- and BR-PDAC. Future studies should consider larger cohorts of the older population, including those with compromised organ functions.

## Figures and Tables

**Table 1:** Comparison of baseline characteristics and neoadjuvant chemoradiation outcomes between the elderly (≥75 years, *n* = 43) and non-elderly (<75 years, *n* = 69) patients.

		Elderly (≥75 Years, *n* = 43)	Non-Elderly (<75 Years, *n* = 69)	*p* Value
Age, year, median (range, IQR)		79 (75–84, 77–81)	68 (44–74, 63–71)	<0.001
Sex, male/female, *n* (%)		22 (51)/21 (49)	43 (62)/26 (38)	0.245
Body mass index, kg/m^2^, median (range, IQR)		22.0 (15.0–27.9, 19.9–23.7)	22.3 (14.0–27.9, 19.9–23.9)	0.818
Hemoglobin level, g/dL, median (range, IQR)		11.1 (7.8–15.5, 10.0–12.6)	11.8 (8.3–15.0, 11.0–13.2)	0.040
Total bilirubin level, mg/dl, median (range, IQR)		0.6 (0.3–1.0, 0.5–0.8)	0.6 (0.2–6.5, 0.5–0.8)	0.690
Performance status *, 0/1, *n* (%)		22 (51)/21 (49)	55 (80)/14 (20)	0.002
Comorbidity, *n* (%)				
	Cardiac disease Chronic kidney disease Diabetes mellitus Pulmonary disease Use of anticoagulants Use of steroid	3 (7) 4 (9) 19 (44) 3 (7) 10 (23) 4 (9)	4 (6) 1 (1) 21 (30) 2 (3) 6 (9) 2 (3)	0.802 0.050 0.140 0.309 0.032 0.143
Preoperative biliary drainage, *n* (%)		22 (51)	47 (68)	0.073
Resectability ^†^, *n* (%)				
	Resectable Borderline resectable	31 (72) 12 (28)	40 (58) 29 (42)	0.131
NACRT protocol, *n* (%)				
	2-week S1 regimen 5-week S1 regimen 2-week Gemcitabine with S1 regimen	15 (35) 15 (35) 13 (30)	27 (39) 24 (35) 18 (26)	0.864
Completion of NACRT protocol, *n* (%)		39 (91)	56 (81)	0.171
	2-week S1 regimen 5-week S1 regimen 2-week Gemcitabine with S1 regimen	15 (100) 13 (87) 11 (85)	23 (85) 19 (79) 14 (78)	0.117 0.553 0.634
Adverse events ^‡^ during NACRT, ≥Grade 3, *n* (%)		10 (23)	12 (17)	0.447
	2-week S1 regimen 5-week S1 regimen 2-week Gemcitabine with S1 regimen	2 (13) 3 (20) 5 (38)	2 (7) 4 (17) 6 (33)	0.531 0.792 0.768
Resection rate, *n* (%)		38 (88)	62 (90)	0.805

* according to the Eastern Cooperative Oncology Group Performance Status, ^†^ according to the National Comprehensive Cancer Network guideline [10], ^‡^ according to the National Cancer Institute Common Terminology Criteria for Adverse Events (CTCAE version 5.0). Abbreviations: IQR, interquartile range; NACRT, neoadjuvant chemoradiotherapy.

**Table 2:** Comparison of complete blood count, renal function, nutritional indices, inflammatory biomarkers, tumor markers, and accumulation of standardized uptake value before and after neoadjuvant chemoradiotherapy between the elderly (≥75 years, *n* = 43) and non-elderly (<75 years, *n* = 69) patients.

Parameter			Elderly (≥75 Years, *n* = 43)	Non-Elderly (<75 Years, *n* = 69)	*p*
Complete blood count, median (range, IQR)					
	White blood cell count,/mm^3^	Pre Post	4990 (2690–11560, 4170–6160) 4220 (2580–8060, 3250–5060)	5670 (2930–12030, 4685–6795) 4110 (1690–10460, 3285–5345)	0.053 0.924
	Neutrophil count,/mm^3^	Pre Post	2911 (1400–8033, 2615–4050) 2420 (1201–6650, 1892–3606)	3613 (1509–9107, 2777–4348) 2810 (989–8954, 2118–3631)	0.044 0.572
	Lymphocyte count,/mm^3^	Pre Post	1238 (668–2750, 1001–1743) 797 (269–2623, 561–1020)	1431 (601–3167, 1221–1779) 879 (169–2370, 603–1134)	0.062 0.446
	Monocyte count,/mm^3^	Pre Post	332 (129–682, 271–384) 329 (132–648, 289–436)	365 (102–1376, 278–427) 350 (106–743, 235–471)	0.100 0.964
	Platelet count, ×10^4^/mm^3^	Pre Post	20.3 (10.1–34.7, 15.7–25.7) 18.3 (7.9–35.5, 13.4–24.0)	23.2 (11.3–43.2, 20.2–26.5) 19.2 (7.9–37.8, 14.1–23.8)	0.006 0.680
eGFR, mL/min./1.73m^2^, median (range, IQR)		Pre Post	74.0 (36.8–112.4, 61.8–81.4) 72.9 (42.2–114.6, 59.0–91.3)	83.4 (47.1–135.5, 70.3–96.9) 84.5 (33.1–144.2, 74.3–100.7)	0.001 0.004
Nutritional indices, median (range, IQR)					
	Albumin level, g/dL	Pre Post	3.6 (2.6–4.6, 3.3–3.9) 3.6 (2.5–4.4, 3.3–3.9)	3.7 (2.3–4.6, 3.5–4.1) 3.7 (1.8–4.9, 3.3–4.0)	0.003 0.373
	Total cholesterol level, mg/dL	Pre Post	166 (21–268, 144–184) 152 (84–259, 126–176)	175 (89–256, 154–204) 161 (98–283, 141–188)	0.082 0.190
	Prognostic nutritional index (PNI)	Pre Post	43.0 (31.0–52.9, 38.3–46.5) 40.8 (28.2-51.1, 35.6-44.4)	46.1 (27.8-56.7, 42.1-49.1) 42.0 (20.3-53.6, 36.9-45.7)	0.017 0.199
Inflammatory biomarkers, median (range, IQR)					
	C-reactive protein, mg/dL	Pre Post	0.20 (0.02-2.82, 0.08-0.46) 0.11 (0.02-3.70, 0.04-0.44)	0.18 (0.01-3.73, 0.08-0.46) 0.14 (0.01-4.54, 0.05-0.39)	0.940 0.495
	Neutrophil-to-lymphocyte ratio (NLR)	Pre Post	2.46 (0.97-7.83, 1.92-2.94) 3.61 (0.92-10.69, 2.33-5.10)	2.37 (0.99-7.83, 1.87-2.98) 3.30 (0.86-16.60, 2.20-4.77)	0.781 0.532
	Platelet-to-lymphocyte ratio (PLR)	Pre Post	151.7 (49.1–405.6, 117.3–199.7) 218.7 (74.0–636.2, 163.4–349.4)	162.8 (57.3–363.1, 134.0–193.3) 211.0 (63.8–1053.3, 161.4–303.0)	0.438 0.528
	Lymphocyte-to-monocyte ratio (LMR)	Pre Post	4.41 (2.25–7.75, 3.31–4.94) 2.21 (0.70–6.31, 1.51–3.29)	4.22 (1.27–12.22, 3.31–5.40) 2.43 (0.76–9.40, 1.96–3.52)	0.863 0.228
	C-reactive protein-to-albumin ratio (CRP/Alb)	Pre Post	0.051 (0.005–1.007, 0.022–0.132) 0.029 (0.005–1.240, 0.012–0.147)	0.044 (0.003–1.243, 0.019–0.136) 0.041 (0.003–1.376, 0.014–0.101)	0.841 0.564
Tumor markers, median (IQR, range)					
	CEA level, ng/ml	Pre Post	3.2 (1.0–56.4, 1.8–4.9) 3.5 (0.9–34.7, 2.3–5.1)	3.6 (0.5–158.3, 2.5–5.4) 3.2 (0.8–11.6, 2.2–4.4)	0.306 0.513
	CA19-9 level, U/mL	Pre Post	140 (2–18159, 66–813) 83 (2–52480, 25–469)	213 (2–70555, 19–813) 68 (2–2500, 10–209)	0.677 0.130
SUVmax level in FDG-PET, median (IQR, range)		Pre Post	6.91 (2.10–30.49, 4.97–10.61) 4.60 (1.44–11.14, 2.79–6.95)	7.55 (2.00–45.71, 4.97–10.61) 4.26 (1.56–47.57, 3.07–6.02)	0.185 0.438

Abbreviations: SUV, standardized uptake value; FDG-PET, fluorodeoxyglucose positron emission tomography; IQR, interquartile range; eGFR, estimated glomerular filtration rate; CEA, carcinoembryonic antigen; CA19-9, carbohydrate antigen 19-9.

**Table 3:** Comparison of clinicopathologic characteristics and perioperative outcomes between the elderly (≥75 years, *n* = 38) and non-elderly (<75 years, *n* = 62) patients who received pancreaticoduodenectomy after neoadjuvant chemoradiotherapy.

		Elderly (≥75 Years, *n* = 38)	Non-Elderly (<75 Years, *n* = 62)	*p*
Operation time, min, median (range, IQR)		499 (357–733, 437–555)	503 (349–816, 468–584)	0.416
Blood loss, ml, median (range, IQR)		1376 (305–6970, 764–2165)	1059 (88–9268, 565–1830)	0.091
Intraoperative transfusion, *n* (%)		20 (53)	19 (31)	0.029
Portal vein resection, *n* (%)		17 (45)	30 (48)	0.723
Mortality, within 90 days, *n* (%)		1 (3)	1 (2)	0.724
Morbidity ^‡^, ≥Grade 3, *n* (%)		11 (29)	8 (13)	0.047
Pancreatic fistula *, Grade B or C, *n* (%)		2 (5)	4 (6)	0.808
Delayed gastric emptying ^†^, Grade B or C, *n* (%)		13 (34)	10 (16)	0.037
Postoperative hospital stay, day, median (range, IQR)		28 (12–203, 16–56)	22 (11–96, 16–32)	0.022
Resection status, R0/R1, *n* (%)		34 (89)/4 (11)	62 (100)/0 (0)	0.009
Node positive pathology, *n* (%)		13 (34)	32 (52)	0.090
Pathological tumor response to NACRT ^§^, *n* (%)				
	Grade I-IIA Grade IIB-IV	24 (63) 14 (37)	38 (56) 26 (42)	0.614
TNM stage ^¶^, *n* (%)				
	0 IA IB IIA IIB III IV	1 (3) 7 (18) 14 (37) 3 (8) 13 (34) 0 (0) 0 (0)	3 (5) 15 (24) 9 (15) 2 (3) 25 (40) 5 (8) 3 (5)	0.064
Postoperative adjuvant chemotherapy received, *n* (%)		27 (71)	52 (84)	0.127
	S-1 Gemcitabine	27 (100) 0 (0)	49 (94) 3 (6)	0.203
Cause of death (*n =* 55 cases)				
	Pancreatic cancer specific Non-pancreatic cancer specific	15 (68) 7 (32)	30 (88) 4 (12)	0.065

* according to the International Study Group of Pancreatic Surgery (ISGPS) classification [19]. ^†^ according to the International Study Group of Pancreatic Surgery (ISGPS) classification [20]. ^‡^ according to Clavien-Dindo classification [16]. ^§^ according to Evans grade classification [21]. ^¶^ according to the Union for International Cancer Control (UICC) TNM classification, 8th edition [17].

**Table 4:** Univariate and multivariate analyses of the clinical variables with regard to overall survival in elderly patients who underwent neoadjuvant chemoradiotherapy followed by pancreatoduodenectomy (*n* = 38).

Variable	Univariate		Multivariate		
	HR	*p*	HR	95% CI	*p*
Monocyte count after NACRT, ≥323/mm^3^	2.37	0.059			
Lymphocyte count after NACRT, <958/mm^3^	3.04	0.024			
Prognostic Nutritional Index after NACRT, ≤36.9	3.09	0.019	3.95	1.06–14.74	0.041
Induction of postoperative adjuvant chemotherapy, no	7.28	<0.001	9.57	2.98–30.68	<0.001
Intraoperative transfusion, yes	2.83	0.018			
Age, ≥80 years	1.17	0.715			

Abbreviations: HR, hazard ratio; CI, confidence interval; NACRT, neoadjuvant chemoradiotherapy.

## Data Availability

The data are available from the corresponding author on reasonable request.

## References

[1] World Health Statistics 2023. https://apps.who.int/iris/bitstream/handle/10665/367912/9789240074323-eng.pdf?sequence=1&isAllowed=y.

[2] Zhu X.H., Wu Y.F., Qiu Y.D., Jiang C.P., Ding Y.T. (2013). Effect of early enteral combined with parenteral nutrition in patients undergoing pancreaticoduodenectomy. World J. Gastroenterol..

[3] Hatzaras I., Schmidt C., Klemanski D., Muscarella P., Melvin W.S., Ellison E.C., Bloomston M. (2011). Pancreatic resection in the octogenarian: A safe option for pancreatic malignancy. J. Am. Coll. Surg..

[4] Khan S., Sclabas G., Lombardo K.R., Sarr M.G., Nagorney D., Kendrick M.L., Donohue J.H., Que F.G., Farnell M.B. (2010). Pancreatoduodenectomy for ductal adenocarcinoma in the very elderly; is it safe and justified?. J. Gastrointest. Surg..

[5] DeOliveira M.L., Winter J.M., Schafer M., Cunningham S.C., Cameron J.L., Yeo C.J., Clavien P.A. (2006). Assessment of complications after pancreatic surgery: A novel grading system applied to 633 patients undergoing pancreaticoduodenectomy. Ann. Surg..

[6] Tani M., Kawai M., Hirono S., Ina S., Miyazawa M., Nishioka R., Shimizu A., Uchiyama K., Yamaue H. (2009). A pancreaticoduodenectomy is acceptable for periampullary tumors in the elderly, even in patients over 80 years of age. J. Hepatobiliary Pancreat. Surg..

[7] Utsumi M., Aoki H., Nagahisa S., Une Y., Kimura Y., Watanabe M., Taniguchi F., Arata T., Katsuda K., Tanakaya K. (2021). Nutritional assessment and surgical outcomes in very elderly patients undergoing pancreaticoduodenectomy: A retrospective study. Surg. Today.

[8] Oguro S., Shimada K., Kishi Y., Nara S., Esaki M., Kosuge T. (2013). Perioperative and long-term outcomes after pancreaticoduodenectomy in elderly patients 80 years of age and older. Langenbecks Arch. Surg..

[9] Belyaev O., Herzog T., Kaya G., Chromik A.M., Meurer K., Uhl W., Müller C.A. (2013). Pancreatic surgery in the very old: Face to face with a challenge of the near future. World J. Surg..

[10] National Comprehensive Cancer Network Clinical Practice Guidelines in Oncology Pancreatic Adenocarcinoma. https://www.nccn.org/professionals/physician_gls/pdf/pancreatic.pdf.

[11] Suto H., Oshima M., Ando Y., Matsukawa H., Takahashi S., Shibata T., Kamada H., Kobara H., Masaki T., Kumamoto K. (2023). Efficacy of neoadjuvant chemoradiotherapy followed by pancreatic resection for older patients with resectable and borderline resectable pancreatic ductal adenocarcinoma. HPB.

[12] Okano K., Suto H., Oshima M., Maeda E., Yamamoto N., Kakinoki K., Kamada H., Masaki T., Takahashi S., Shibata T. (2017). A Prospective Phase II Trial of Neoadjuvant S-1 with Concurrent Hypofractionated Radiotherapy in Patients with Resectable and Borderline Resectable Pancreatic Ductal Adenocarcinoma. Ann. Surg. Oncol..

[13] Suto H., Okano K., Oshima M., Ando Y., Matsukawa H., Takahashi S., Shibata T., Kamada H., Kobara H., Tsuji A. (2022). Efficacy and Safety of Neoadjuvant Chemoradiation Therapy Administered for 5 Versus 2 Weeks for Resectable and Borderline Resectable Pancreatic Cancer. Pancreas.

[14] Oettle H., Neuhaus P., Hochhaus A., Hartmann J.T., Gellert K., Ridwelski K., Niedergethmann M., Zulke C., Fahlke J., Arning M.B. (2013). Adjuvant chemotherapy with gemcitabine and long-term outcomes among patients with resected pancreatic cancer: The CONKO-001 randomized trial. JAMA.

[15] Uesaka K., Boku N., Fukutomi A., Okamura Y., Konishi M., Matsumoto I., Kaneoka Y., Shimizu Y., Nakamori S., Sakamoto H. (2016). Adjuvant chemotherapy of S-1 versus gemcitabine for resected pancreatic cancer: A phase 3, open-label, randomised, non-inferiority trial (JASPAC 01). Lancet.

[16] Clavien P.A., Barkun J., de Oliveira M.L., Vauthey J.N., Dindo D., Schulick R.D., de Santibanes E., Pekolj J., Slankamenac K., Bassi C. (2009). The Clavien-Dindo classification of surgical complications: Five-year experience. Ann. Surg..

[17] Brierley J.D., Gospodarowicz M.K., Wittekind C. (2016). TNM Classification of Malignant Tumours.

[18] Fluss R., Faraggi D., Reiser B. (2005). Estimation of the Youden Index and its associated cutoff point. Biom. J..

[19] Bassi C., Marchegiani G., Dervenis C., Sarr M., Abu Hilal M., Adham M., Allen P., Andersson R., Asbun H.J., Besselink M.G. (2017). The 2016 update of the International Study Group (ISGPS) definition and grading of postoperative pancreatic fistula: 11 Years After. Surgery.

[20] Wente M.N., Bassi C., Dervenis C., Fingerhut A., Gouma D.J., Izbicki J.R., Neoptolemos J.P., Padbury R.T., Sarr M.G., Traverso L.W. (2007). Delayed gastric emptying (DGE) after pancreatic surgery: A suggested definition by the International Study Group of Pancreatic Surgery (ISGPS). Surgery.

[21] Evans D.B., Rich T.A., Byrd D.R., Cleary K.R., Connelly J.H., Levin B., Charnsangavej C., Fenoglio C.J., Ames F.C. (1992). Preoperative chemoradiation and pancreaticoduodenectomy for adenocarcinoma of the pancreas. Arch. Surg..

[22] Takahashi S., Ohno I., Ikeda M., Konishi M., Kobayashi T., Akimoto T., Kojima M., Morinaga S., Toyama H., Shimizu Y. (2022). Neoadjuvant S-1 With Concurrent Radiotherapy Followed by Surgery for Borderline Resectable Pancreatic Cancer: A Phase II Open-label Multicenter Prospective Trial (JASPAC05). Ann. Surg..

[23] Katz M.H., Shi Q., Ahmad S.A., Herman J.M., Marsh Rde W., Collisson E., Schwartz L., Frankel W., Martin R., Conway W. (2016). Preoperative Modified FOLFIRINOX Treatment Followed by Capecitabine-Based Chemoradiation for Borderline Resectable Pancreatic Cancer: Alliance for Clinical Trials in Oncology Trial A021101. JAMA Surg..

[24] Murphy J.E., Wo J.Y., Ryan D.P., Jiang W., Yeap B.Y., Drapek L.C., Blaszkowsky L.S., Kwak E.L., Allen J.N., Clark J.W. (2018). Total neoadjuvant therapy with FOLFIRINOX followed by individualized chemoradiotherapy for borderline resectable pancreatic adenocarcinoma: A phase 2 clinical trial. JAMA Oncol..

[25] Motoi F., Kosuge T., Ueno H., Yamaue H., Satoi S., Sho M., Honda G., Matsumoto I., Wada K., Furuse J. (2019). Randomized phase II/III trial of neoadjuvant chemotherapy with gemcitabine and S-1 versus upfront surgery for resectable pancreatic cancer (Prep-02/JSAP05). Jpn. J. Clin. Oncol..

[26] Sulpice L., Rayar M., D’Halluin P.N., Harnoy Y., Merdrignac A., Bretagne J.F., Meunier B., Boudjema K. (2012). Impact of age over 75 years on outcomes after pancreaticoduodenectomy. J. Surg. Res..

[27] Ballarin R., Spaggiari M., Di Benedetto F., Montalti R., Masetti M., De Ruvo N., Romano A., Guerrini G.P., De Blasiis M.G., Gerunda G.E. (2009). Do not deny pancreatic resection to elderly patients. J. Gastrointest. Surg..

[28] Okadome K., Baba Y., Yagi T., Kiyozumi Y., Ishimoto T., Iwatsuki M., Miyamoto Y., Yoshida N., Watanabe M., Baba H. (2020). Prognostic Nutritional Index, Tumor-infiltrating Lymphocytes, and Prognosis in Patients with Esophageal Cancer. Ann. Surg..

[29] Abe T., Nakata K., Kibe S., Mori Y., Miyasaka Y., Ohuchida K., Ohtsuka T., Oda Y., Nakamura M. (2018). Prognostic Value of Preoperative Nutritional and Immunological Factors in Patients with Pancreatic Ductal Adenocarcinoma. Ann. Surg. Oncol..

[30] Itoh S., Tsujita E., Fukuzawa K., Sugimachi K., Iguchi T., Ninomiya M., Maeda T., Kajiyama K., Adachi E., Uchiyama H. (2021). Prognostic significance of preoperative PNI and CA19-9 for pancreatic ductal adenocarcinoma: A multi-institutional retrospective study. Pancreatology.

[31] van Dam R.M., Hendry P.O., Coolsen M.M., Bemelmans M.H., Lassen K., Revhaug A., Fearon K.C., Garden O.J., Dejong C.H., Enhanced Recovery After Surgery (ERAS) Group (2008). Initial experience with a multimodal enhanced recovery programme in patients undergoing liver resection. Br. J. Surg..

[32] Xu X., Zheng C., Zhao Y., Chen W., Huang Y. (2018). Enhanced recovery after surgery for pancreaticoduodenectomy: Review of current evidence and trends. Int. J. Surg..

[33] Balzano G., Zerbi A., Braga M., Rocchetti S., Beneduce A.A., Di Carlo V. (2008). Fast-track recovery programme after pancreaticoduodenectomy reduces delayed gastric emptying. Br. J. Surg..

